# Coordinated Gene Expression of Neuroinflammatory and Cell Signaling Markers in Dorsolateral Prefrontal Cortex during Human Brain Development and Aging

**DOI:** 10.1371/journal.pone.0110972

**Published:** 2014-10-20

**Authors:** Christopher T. Primiani, Veronica H. Ryan, Jagadeesh S. Rao, Margaret C. Cam, Kwangmi Ahn, Hiren R. Modi, Stanley I. Rapoport

**Affiliations:** 1 Brain Physiology and Metabolism Section, Laboratory of Neurosciences, National Institute on Aging, National Institutes of Health, Bethesda, Maryland, United States of America; 2 Office of Science and Technology Resources, National Cancer Institute, National Institutes of Health, Bethesda, Maryland, United States of America; 3 Child Psychiatry Branch, National Institute of Mental Health, National Institutes of Health, Bethesda, Maryland, United States of America; University of Wuerzburg, Germany

## Abstract

**Background:**

Age changes in expression of inflammatory, synaptic, and neurotrophic genes are not well characterized during human brain development and senescence. Knowing these changes may elucidate structural, metabolic, and functional brain processes over the lifespan, as well vulnerability to neurodevelopmental or neurodegenerative diseases.

**Hypothesis:**

Expression levels of inflammatory, synaptic, and neurotrophic genes in the human brain are coordinated over the lifespan and underlie changes in phenotypic networks or cascades.

**Methods:**

We used a large-scale microarray dataset from human prefrontal cortex, BrainCloud, to quantify age changes over the lifespan, divided into Development (0 to 21 years, 87 brains) and Aging (22 to 78 years, 144 brains) intervals, in transcription levels of 39 genes.

**Results:**

Gene expression levels followed different trajectories over the lifespan. Many changes were intercorrelated within three similar groups or clusters of genes during both Development and Aging, despite different roles of the gene products in the two intervals. During Development, changes were related to reported neuronal loss, dendritic growth and pruning, and microglial events; *TLR4*, *IL1R1*, *NFKB1*, *MOBP*, *PLA2G4A*, and *PTGS2* expression increased in the first years of life, while expression of synaptic genes *GAP43* and *DBN1* decreased, before reaching plateaus. During Aging, expression was upregulated for potentially pro-inflammatory genes such as *NFKB1*, *TRAF6*, *TLR4*, *IL1R1*, *TSPO*, and *GFAP*, but downregulated for neurotrophic and synaptic integrity genes such as *BDNF*, *NGF*, *PDGFA*, *SYN*, and *DBN1*.

**Conclusions:**

Coordinated changes in gene transcription cascades underlie changes in synaptic, neurotrophic, and inflammatory phenotypic networks during brain Development and Aging. Early postnatal expression changes relate to neuronal, glial, and myelin growth and synaptic pruning events, while late Aging is associated with pro-inflammatory and synaptic loss changes. Thus, comparable transcriptional regulatory networks that operate throughout the lifespan underlie different phenotypic processes during Aging compared to Development.

## Introduction

The human brain changes structurally, functionally, and metabolically throughout the lifespan [1], [2]. Programmed dendritic growth followed by pruning, neuronal loss, shifts in energy metabolism from ketone body to glucose consumption, and rapid myelination occur during development [3], [4], [5], [6]. Many of these changes are completed within the first two decades of life, although myelination can continue into the fourth decade [5], [7], [8]. In middle age, the brain reaches a level of homeostatic stability, but neuronal and synaptic loss associated with cognitive changes can appear later on [2], [9], [10], [11], [12]. Aging also is a risk factor for progressive brain disorders in which neuroinflammation plays a prominent role [13], [14], [15], [16], [17].

Neuroinflammation involves activation of resident brain microglia and astrocytes, and can be produced by different internal or external stresses [14], [18], [19], [20]. Microglial activation *via* toll-like receptors (TLRs) or cluster of differentiation (CD)14 receptors releases cytokines such as interleukin (IL)-1β, IL-6, tumor necrosis factor-α (TNFα) and interferon gamma (IFNγ), chemokines such as fractalkine (CX3CL1), and nitric oxide (NO), following activation of inducible nitric oxide synthase (iNOS), thereby creating response cascades that can negatively impact brain structure and function [21]. Downstream activation of IL-1 receptors (IL-1R) and TNFα receptors on astrocytes and other cell types alters levels of transcription factors such as nuclear factor-kappa B (NF-κB) and activator protein (AP)-2, which can increase expression of a number of inflammatory genes [21], [22], [23], [24], [25].

The term “inflamm-aging” has been proposed to describe the progressive increase in proinflammatory status in the brain with senescence [26]. Inflamm-aging may prime brain microglia and astrocytes to respond excessively to different stressors, including neurodegenerative, traumatic or infectious insults [27], [28], [29], [30], [31]. Increased inflammatory response markers with late-state brain aging have been documented in rodents [27], [32], nonhuman primates [33], and humans [34]. Increases have been noted in proinflammatory cytokines IL-1α, IL-1β, IL-18 and IFNγ, major histocompatibility complex class II (MHC II), CD11b, scavenger receptors CD68, CD86 and CD40, and TLRs 1, 2, 4, 5, 7, and 9. In the human brain, increased TNFα and interferon gamma-inducible protein 16 (IFI-16) were reported, as were increased mRNA and protein levels of CD11b, glial fibrillary associated protein (GFAP), IL-1β, iNOS, NF-κB p50, cytosolic phospholipase A_2_ (cPLA_2_) Type IVA and cyclooxygenase (COX)-2, while levels of brain derived neurotropic factor protein (BDNF) and synaptophysin (SYP) were reduced [34], [35].

Cytokines, chemokines, growth and other microglial and astrocytic factors that change with age in the adult brain also have important regulatory actions during neurodevelopment [36], [37]. For example, microglia participate postnatally in synaptic pruning and apoptosis, and produce nerve growth factor (NGF), BDNF, neurotrophin (NT)-3 and cytokines that influence neuronal path finding, synaptogenesis and experience dependent plasticity [38], [39], [40], [41].

Multiple metabolic and protein networks have been described that underlie brain structure and function, and brain vulnerability to disease [42], [43], [44], [45], [46]. The extent to which these phenotypic networks or “cascades” are regulated at the transcriptional level, particularly during brain development, maturity, and aging are not well understood. To address this limitation, in the present study we analyzed age changes over the lifespan in brain mRNA levels of 39 genes whose protein products have been reported to be involved in neuroinflammation, synaptic integrity, neurotrophic effects, and related processes. As in our prior report on age expression of brain lipid metabolic markers [47], we employed the large-scale microarray dataset called BrainCloud, which contains genome-wide expression levels in frontal cortex from non-pathological individuals, in the fetal period to postnatal 78 years of age [48]. Similarly, and consistent with the literature, we considered gene expression in two distinct postnatal age intervals, Development (0 to 21 years) and Aging (22 to 78 years) [35], [47], [49], [50], henceforth identified by capitalizations.

Based on prior studies, we hypothesized that expression of genes linked to neuroinflammation would increase with age in the Aging interval [29], [34], [51], [52], while expression of genes coding for synaptic integrity and plasticity would decrease [9]. Furthermore, expression of these same genes would change during Development to reflect reported roles of their protein products in synaptic and neuronal growth, pruning, myelination, and other events in this period [1].

We also hypothesized that expression of genes coding for products that belong to common growth and neuroinflammatory cascades would be coordinately regulated during the lifespan, in relation to the specific phenotypic networks in which their proteins interacted. Coordinated or synchronized gene transcription underlying changes in phenotype networks has been demonstrated in cell culture, rodent brain, and artificial systems [53], [54], [55], and in the human brain in relation to age [47], [48], [56], [57], [58].

## Methods

### BrainCloud

BrainCloud (http://braincloud.jhmi.edu/) is a publically available software program that contains gene expression and methylation microarray datasets of more than 30,000 probes [48]. We analyzed data from 231 dorsolateral prefrontal cortex samples (Brodmann Areas 46/49) from subjects ranging in age from birth to 78 years. Results were transferred to an accessible and easily operated interface. Subjects had no history of significant psychiatric disorder, as determined by telephone screening and medical examiner reports [48]. Cause of death was listed as accident, homicide, or natural cause. Toxicology measurements were taken post-mortem and history of drug abuse and neuropathology tests were assessed [48]. Samples were genotyped with Illumina Infinium HD Gemini 1M Duo BeadChips or with Illumina Infinium II 650 K. mRNA was quantified with the Illumina Human 49K Oligo array (HEEBO-7 set) [48].

Based on the literature and to remain consistent with our prior publication [35], [47], [49], [50], we divided our sample into a Development interval (0 to 21 years, n = 87, mean age: 10.99) and an Aging interval (22 to 78 years, n = 144, mean age: 45.38), capitalized to distinguish these defined intervals from the developmental and aging processes themselves. The brains were from 73 females and 158 males.

When multiple probes in BrainCloud represented a gene, we selected the probe sequence that covered all exon-coding regions of the gene's identified transcripts. The highest intensity probe (average log_2_ intensity of fluorescent signal for this probe across all subjects) was taken when two probes covered all exon-coding regions of the transcripts. Most probes that met the first criterion also were the highest intensity probes of that gene. Genes were selected based on their availability in the BrainCloud dataset. Gene expression levels remained consistent with BrainCloud's calculated expression as log_2_ (Sample/Reference), reference being the pooled RNA from all subjects [48].

### Statistical Analyses

Pearson's r correlations were used to correlate expression levels of pairs of genes. Linear regression was used to relate expression to age within the separate Development and Aging intervals. Nonlinear best-fit comparisons with each gene were performed using ‘The extra sum-of-squares F test’ to compare one-phase decay to a first-order linear model. A four-way ANOVA that included relevant factors provided by BrainCloud software, such as gender, race and batch number, was performed to calculate fold-differences and p-values comparing the Aging with Development group. The p-values from the ANOVA results include correction of significance for multiple comparisons using the Benjamini-Hochberg [59] control of False Discovery Rate (FDR), also known as the “step-up” FDR procedure. Batch effect removal on factors (array batch, race, and sex) was performed using the sva package (version 3.8.0) in Bioconductor, based on the method of Johnson et al. [60]. No other normalization was performed on the raw data. Differential gene expression would be a result of age and not race, sex, or array batch. Pearson's r correlations and multiple ANOVAs were performed in Partek Genomics Suite (Version 6.6, Partek, St. Louis, MO, USA). Linear regression analyses and scatter plots were performed using GraphPad Prism version 6.0 for Mac OS X (GraphPad Software, La Jolla, CA, USA, www.graphpad.com).

### Heat Map

Heat maps, which are graphical representations of data where individual values are represented in a matrix on a color scale, were created using Partek Genomics Suite 6.6 to visualize correlated expression levels across all genes in the separate Development and Aging periods.

## Results

### Differences in Gene Expression during Development and Aging

#### Correlations with age

**Figure 1:**
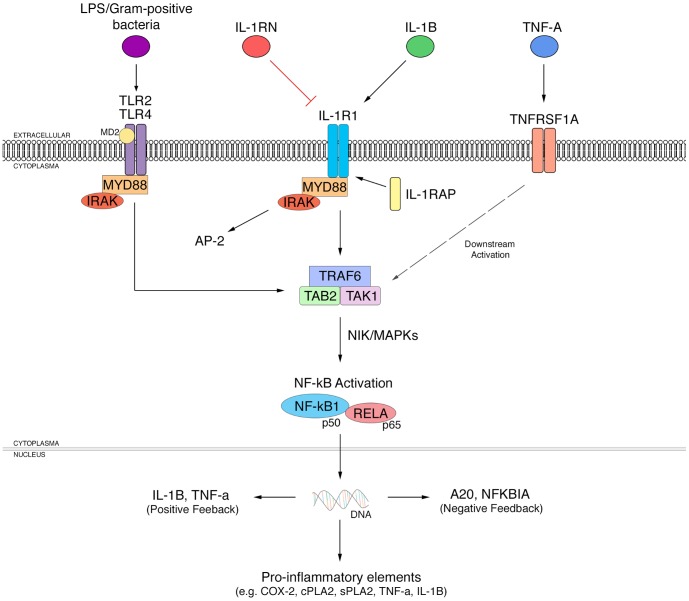
Pathways associated with activation of toll-like receptors TLR-2 and TLR-4, the IL-1 receptor IL-1R1, and the TNFα receptor, TNFRSF1A. IL-1β binding to the IL-1R1 leads to recruitment of IL-1 receptor accessory protein (IL-1RAP), and can be blocked by the naturally occurring IL-1 receptor antagonist, IL-1RN. The IL-1R1/1β/RAP signaling complex is capable of recruiting interleukin receptor-associated kinase (IRAK), IL-1 receptor accessory protein (IL-1RAP), and myeloid differentiation factor 88 (MYD88). IRAK can be phosphorylated and subsequently dissociate from the receptor complex to interact with tumor necrosis factor receptor-associated factor (TRAF6) and TGF-β activated kinase 1 (TAK1)/MAP3K7 binding protein 2 (TAB2) complex. The TLR2 and TLR4 cascades are simplified in Figure 1 into a single cascade. However, gram-negative bacterial lipopolysaccharide (LPS) can activate TLR4, which associates with lymphocyte antigen-96 (MD-2) while gram-positive bacteria are recognized by TLR2 [115]. Their activation effects converge with those of the IL-1R and TNFα receptor on the TRAF6 complex. IL-1R and all TLRs except TLR3 exhibit the same Toll/interleukin-1 receptor (TIR) region that allows recruitment of MYD88 upon activation [116]. Nuclear I kappa kinase (NIK) and various mitogen-activated protein kinases (MAPKs) can promote activation of cytoplasmic nuclear factor-kappa B (NF-κB). Activated NF-κB then can enter the nucleus of the cell to regulate transcription of various genes by binding to their promoter regions. RELA (p65) and p50, proteins in NF-κB family; AP-2: transcription factor AP-2 alpha (activating enhancer binding protein 2 alpha); A20: tumor necrosis factor, alpha-induced protein 3; NFKBIA: nuclear factor of kappa light polypeptide gene enhancer in B-cells inhibitor, alpha; COX-2: cyclooxygenase-2; cPLA_2_: phospholipase A_2_ (cytosolic, calcium-dependent); sPLA_2_: phospholipase A_2_ (secretory).

Table S1 presents the 39 genes that were selected in this study, with their corresponding chromosomal location, protein name, and reported general functions [61], [62], [63], [64]. While many of their protein functions may be unrelated, many of their proteins have been reported to participate in phenotypic cascades involving cell growth, inflammation, synaptic maintenance and gene transcription, and other processes. For example, Figure 1, generated from the literature [65], [66], [67], [68], [69] and string interactions in GeneCards [64], illustrates several abbreviated cascades initiated by activation of TLR-2 and TLR-4 commonly found on microglia, and of IL-1R and TNFα receptors commonly found on neurons and astrocytes. These cascades are abnormal in a number of progressive neuroinflammatory diseases [13], [15], [69], [70], [71].

**Figure 2:**
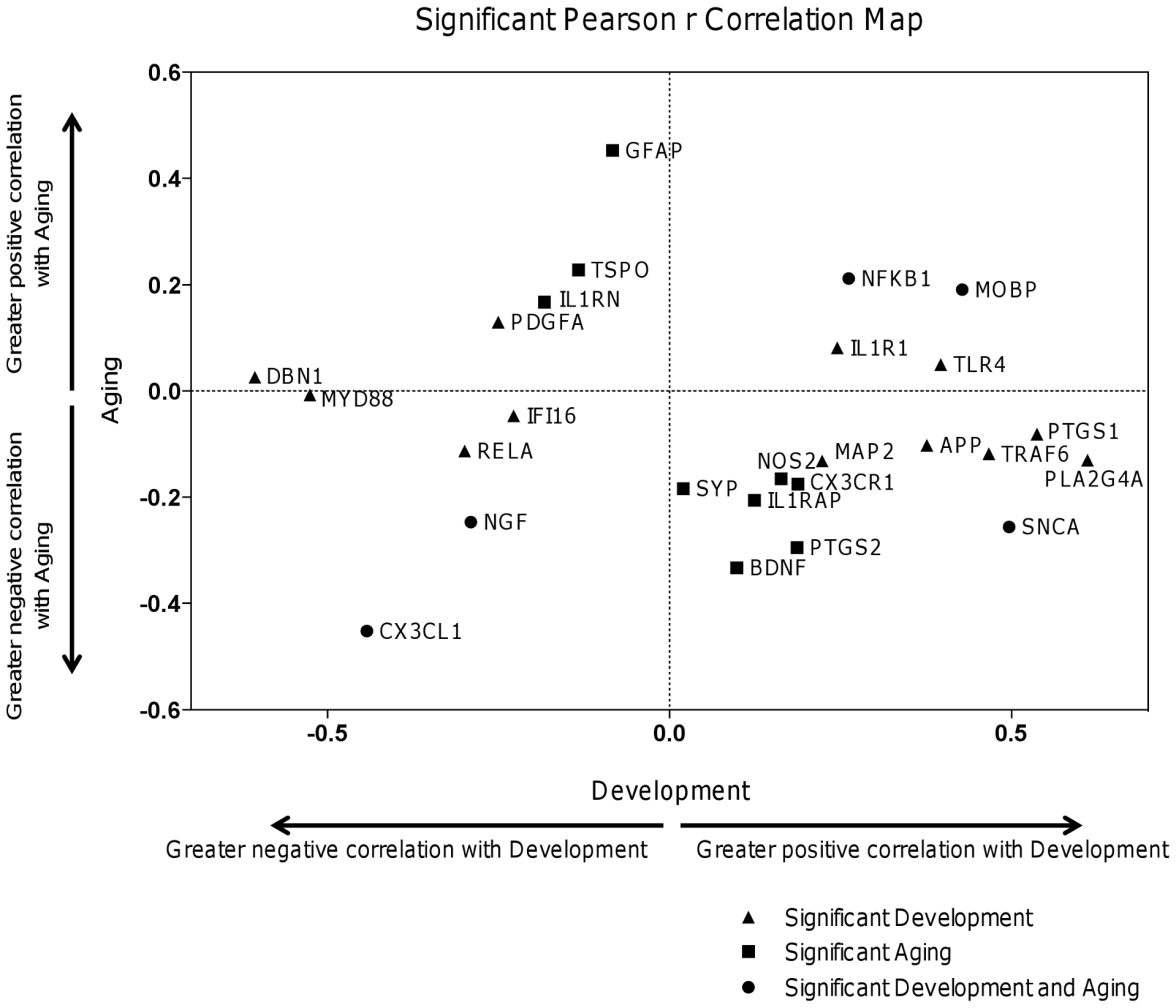
Statistically significant correlations with age in Development and/or Aging intervals. Graphical identification of genes with statistically significant (p<0.05) Pearson r correlations in expression level during Development (x axis) and Aging (y axis) intervals. Gene expressions negatively correlated with age during Development are to the left, while genes positively correlated are to the right of the vertical line. Genes that were negatively correlated with age in the Aging group are below the horizontal line, while genes positively correlated are above line. Development: n = 87; Aging: n = 144.

Of the genes listed in Table S1, Figure 2 summarizes those whose expression levels correlated significantly with age in the Development and/or Aging interval, and indicates by quadrant the direction of the correlation. Age-related expression of *MOBP* (myelin-associated oligodendrocyte basic protein) and of *NFKB1* increased during both Development and Aging (right upper quadrant), while expression of *NGF* and *CX3CL1* (Chemokine (C-X3-C Motif) Ligand 1) decreased during both Development and Aging (left lower quadrant). Expression of *SNCA*, coding for α-synuclein, increased during Development but decreased during Aging (right lower quadrant). Additionally, expression of *TRAF6* (TNF receptor associated factor 6), *PLA2G4A*, *PTGS1*, *TLR4*, *APP*, *MAP2*, and *IL1R1* increased while expression of *MYD88*, *DBN1*, *RELA*, *PDGFA*, and *IFI16* decreased during Development but not Aging. Conversely, expression of *GFAP*, *TSPO*, and *IL1RN* increased and of *PTGS2*, *BDNF*, *IL1RAP*, *SYP*, *CX3CR1*, and *NOS2* decreased during Aging, without changing significantly with age during the Development interval.

**Figure 3:**
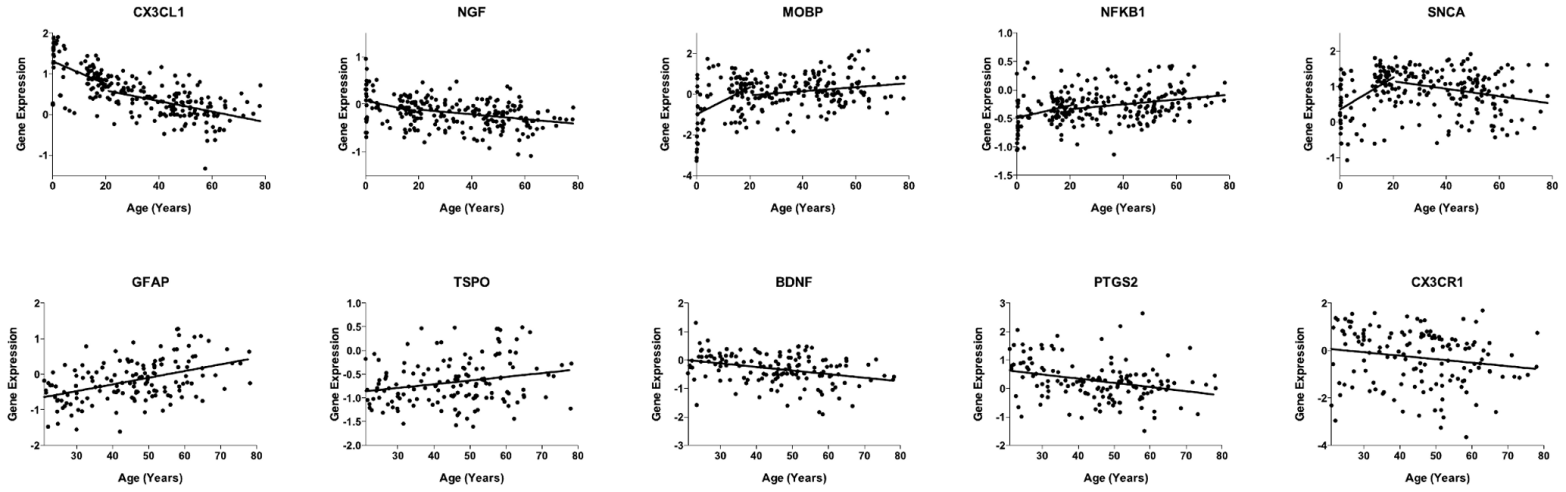
Significant linear regressions of gene expression during both Development and Aging intervals (top), and Aging interval alone (bottom). Scatterplots illustrating log_2_ gene expression over age in years. An increase or decrease of 1 on the log_2_ scale (y-axis) represents a two-fold change in gene expression in the positive or negative direction, respectively. Each data point represents observation from one brain (Development: n = 87; Aging: n = 144). Gene name (p-value during Development, p-value during Aging) - *CX3CR1* (p<0.0001, p<0.0001), *NGF* (p = 0.006, p = 0.002), *MOBP* (p<0.0001, p = 0.02), *NFκB1* (p = 0.01, p = 0.01), SNCA (p<0.0001, p = 0.002). Genes significant in only Aging interval – *GFAP* (p<0.0001), *TSPO* (p = 0.006), *BDNF* (p<0.0001), *PTGS2* (p = 0.0003), *CX3CR1* (p = 0.03).

Figure 3 gives examples of genes whose expression levels correlated significantly with age during both the Development and Aging periods. Expression of *SNCA*, increased significantly with age during Development while declining during Aging. Expression of *MOBP* and *NFKB1* increased significantly with age during both intervals, while expression *CX3CL1* and *NGF* decreased. Expression of *CX3CR1*, and *PTGS2* (COX-2), declined with age whereas *GFAP* and *TSPO* increased during both intervals.

**Figure 4:**
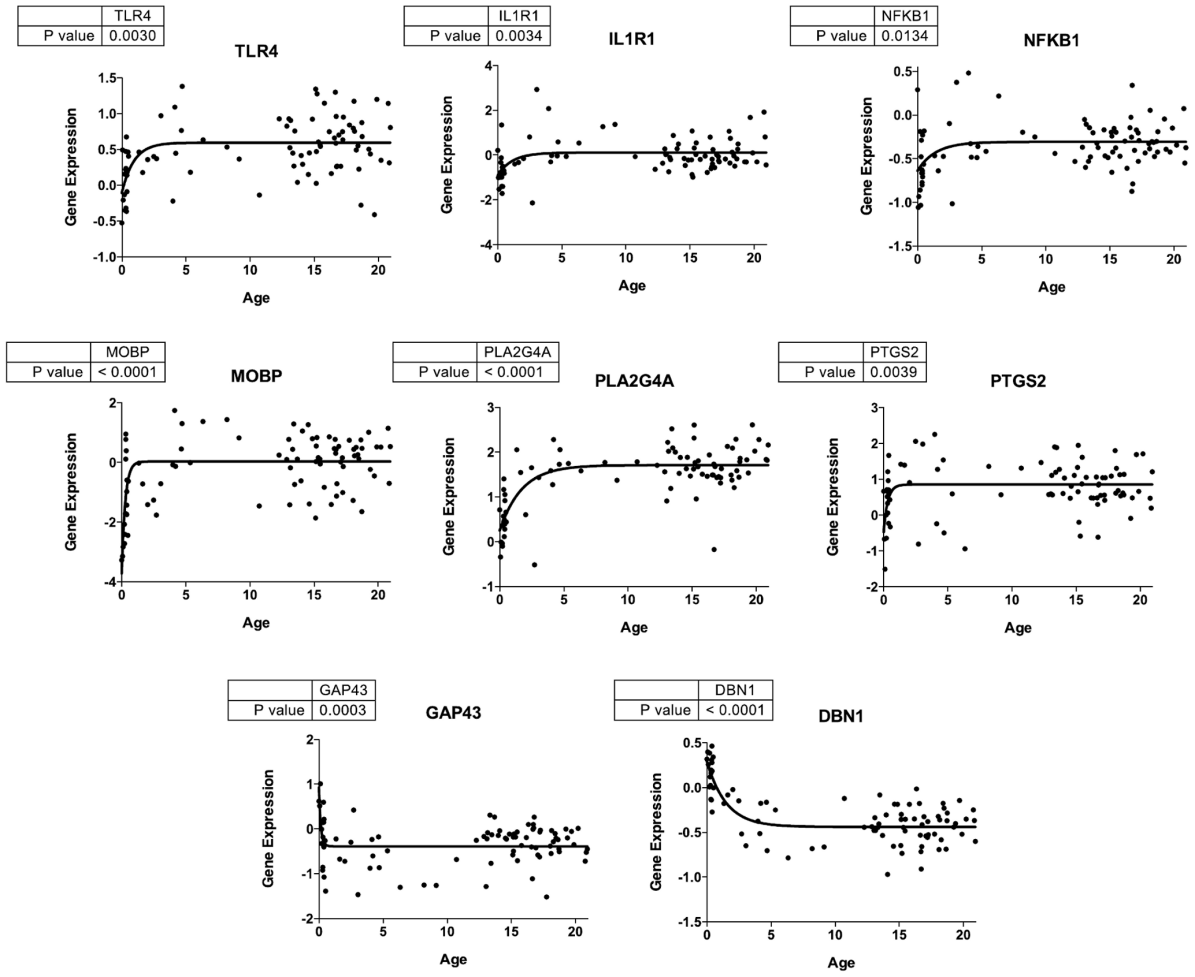
Nonlinear fits for expression levels with age of eight genes during Development. Fitted line added to expression data following equation for 0 to 21 years, Y = (Y0−Plateau)*exp(−K*A)+Plateau, where Y = expression level at age A, and Y0 expression level at A = 0 years). An increase or decrease of 1 on the log_2_ scale (y-axis) represents a two-fold change in expression in the positive or negative direction, respectively.

Visual observation of several gene expression levels during Development suggested non-linearity, with initial levels in the first year of life being higher or lower than later plateaus. To test this, we compared goodness of fit with a non-linear equation, Y = (Y0−Plateau)*exp(−K*A)+Plateau (where Y = expression level at age A, and Y0 = expression level at A = 0 years) to that of a linear regression during Development for each gene studied. As illustrated in Figure 4, expression of *TLR4*, *IL1R1*, *NFKB1*, *MOBP*, *PLA2G4A*, and *PTGS2* increased in the first years of life and reached a plateau, while expression of synaptic genes *GAP43* and *DBN1* decreased before reaching a plateau.

### Mean expression level differences between Aging and Developmental Periods

As summarized in Table 1, mean expression levels were significantly (adjusted p<0.05) lower during Aging than Development for *CX3CR1*, *CX3CL1*, *PTGS2*, *BDNF*, *CASP1*, *CD68*, *AIF1*, *MYD88*, *NGF*, *PDGFA*, *DBN1*, *IL1B*, and *SYP*, and significantly higher for *GFAP*, *TSPO*, *MOBP*, *TRAF6*, and *NFKB1* (*highlights show significance after correction for multiple comparisons*).

**Table 1:** Statistically significant mean gene expression differences between Aging and Development Periods (Multiple ANOVA results).

Reduced with Aging			Increased with Aging		
Gene	Adjusted p-value	Fold-Difference	Gene	Adjusted p-value	Fold-Difference
*CX3CL1*	5.07E-21	−1.75	*GFAP*	4.33E-05	1.37
*CX3CR1*	8.00E-06	−2.00	*TSPO*	1.87E-03	1.23
*BDNF*	1.53E-05	−1.39	*MOBP*	2.77E-03	1.43
*CD68*	1.56E-04	−1.26	*TRAF6*	3.41E-03	1.17
*CASP1*	1.63E-04	−1.35	*NFKB1*	7.76E-03	1.11
*AIF1* (IBA1)	1.63E-04	−1.31	*TLR4*	*6.16E-02*	*1.12*
*PTGS2* (COX-2)	1.75E-04	−1.44	*IL1R1*	*9.70E-02*	*1.21*
*PDGFA*	1.34E-03	−1.14			
*DBN1*	1.34E-03	−1.13			
*MYD88*	1.34E-03	−1.24			
*NGF*	1.34E-03	−1.14			
*SYP*	2.11E-03	−1.18			
*IL1B*	7.33E-03	−1.22			
*BACE1*	*5.33E-02*	*−1.11*			

Multiple ANOVA results, giving fold changes, showing that mean expression was significantly lower (left) or higher (right) in Aging compared with Development. A negative fold-change represents decreased expression in the Aging compared to Development group, and *vice versa*. Italicized p-values are nonsignificant after correction for multiple comparisons. In p-values, term E-number = ×10^−number^. (Development: n = 87; Aging: n = 144).

### Correlated group expression changes during Development and Aging

**Figure 5:**
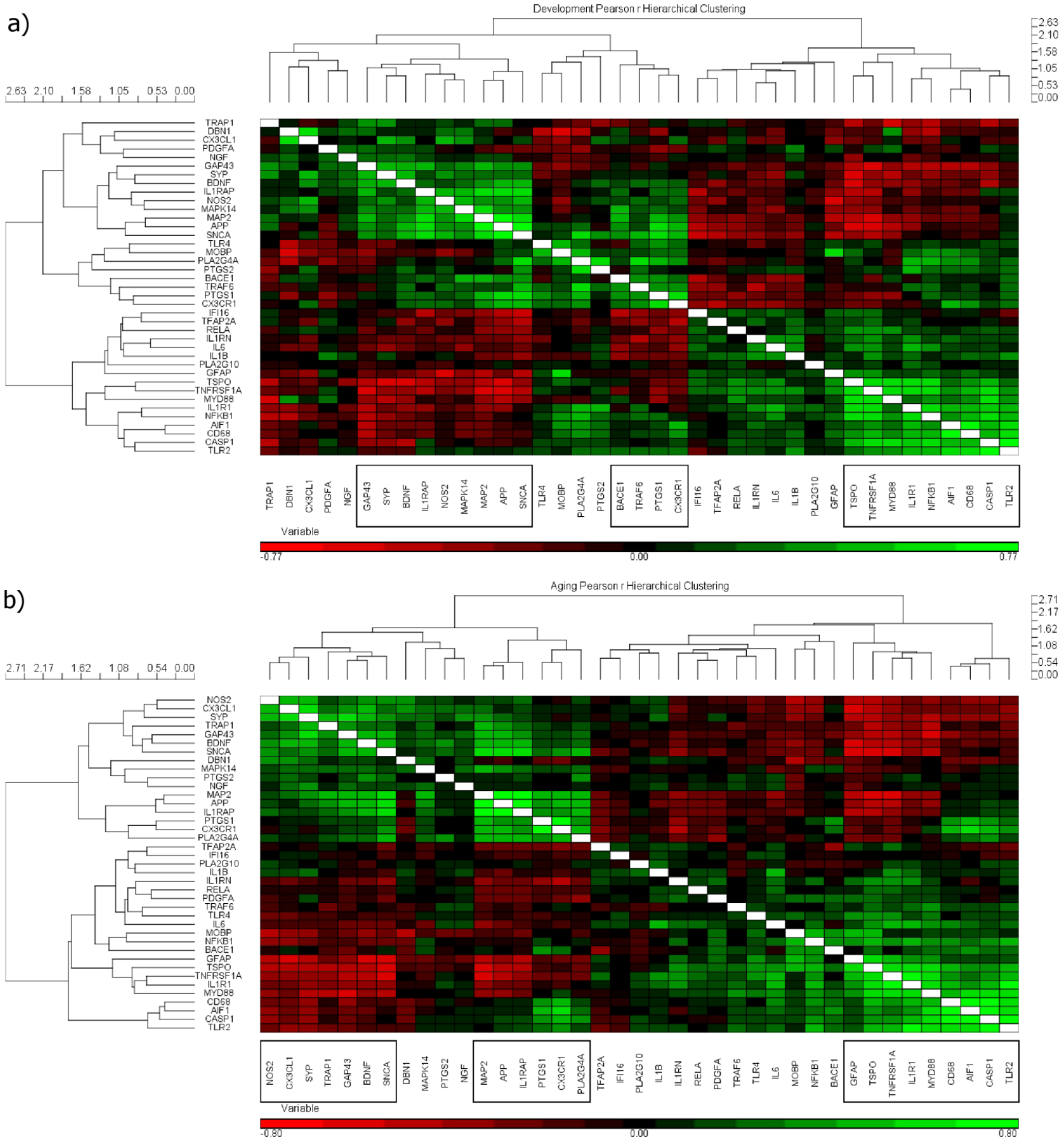
Similarity matrices (hierarchically clustered heat maps) of Pearson's r correlations of gene expression levels with age in Development (A) and Aging (B) groups. Red indicates negatively correlated associations; green are positively correlated associations, while black represents non-significant associations between gene pairs. Genes are clustered hierarchically along the left y-axis, which is mirrored above in each heat map.

Pearson's correlation matrices relating all combinations of the genes were visualized using unsupervised hierarchical clustering and heat maps within the Development (Figure 5a) and Aging (Figure 5b) intervals. Gene order based on hierarchical clustering are not conserved between Development and Aging heat maps, as they represent the highest probability of correctly clustering genes based on Pearson's r correlation in the individual intervals. In Figures 5a and 5b, genes that are highly positively correlated within a cluster are highlighted in green; those that are negatively correlated in red.

Three different clusters of genes whose expression levels were highly intercorrelated were identified in both the Development and Aging periods. As illustrated in Figure 5a, Development clusters were: Cluster 1: *GAP43*, *SYP*, *BDNF*, *IL1RAP*, *NOS2*, *MAPK14*, *MAP2*, *APP*, *SNCA*; Cluster 2: *BACE1*, *TRAF6*, *PTGS1*, *CX3CR1*; Cluster 3: *TSPO*, *TNFRSF1A*, *MYD88*, *IL1R1*, *NFKB1*, *AIF1*, *CD68*, *CASP1*, *TLR2*. As illustrated in Figure 5b, Aging clusters were: Cluster 1: *NOS2*, *CX3CL1*, *SYP*, *TRAP1*, *GAP43*, *BDNF*, *SNCA*; Cluster 2: *MAP2*, *APP*, *IL1RAP*, *PTGS1*, *CX3CR1*, *PLA2G4A*; Cluster 3: *GFAP*, *TSPO*, *TNFRSF1A*, *IL1R1*, *MYD88*, *CD68*, *AIF1*, *CASP1*, *TLR2*.

Figures S1a and S1b show pairwise correlation values between gene expression levels in the Development and Aging periods, respectively. Green coloring highlights statistical significance at p≤0.0001. The global clusters identified in Figures 5a and 5b, and the highly significant pairwise correlations illustrated in Figures S1a and S1b, show plausible synchronization of gene transcription within clusters or networks throughout the lifespan. The most significant pairwise correlations taken from Figures S1a and S1b, at p<10^−10^ and r≥|0.6|, are given in Table 2.

**Table 2:** Highly significant (r≥|0.6|) pair-wise correlations in age-related gene expression during Development and Aging intervals.

Development				Aging			
Gene1	Gene2	Correlation	p-value	Gene1	Gene2	Correlation	p-value
*MAP2*	*APP*	0.77	1.53E-18	*MAP2*	*APP*	0.80	5.61E-34
*AIF1*	*CD68*	0.75	3.86E-17	*IL1R1*	*TNFRSF1A*	0.76	3.38E-29
*TLR2*	*CASP1*	0.67	8.13E-13	*AIF1*	*CD68*	0.75	1.49E-27
*APP*	*SNCA*	0.67	9.95E-13	*AIF1*	*CASP1*	0.71	4.82E-24
*MOBP*	*DBN1*	-0.63	3.20E-11	*TLR2*	*CASP1*	0.69	1.84E-22
*MAP2*	*SNCA*	0.63	3.37E-11	*CD68*	*CASP1*	0.68	1.54E-21
*TSPO*	*MYD88*	0.63	5.75E-11	*CX3CR1*	*PTGS1*	0.68	1.68E-21
*IL1R1*	*NFKB1*	0.61	2.88E-10	*TLR2*	*IL1R1*	0.68	3.63E-21
*CX3CR1*	*PTGS1*	0.60	4.02E-10	*IL1RAP*	*APP*	0.67	1.25E-20
*MYD88*	*CASP1*	0.60	6.18E-10	*IL1RAP*	*MAP2*	0.67	1.87E-20
*IL1R1*	*TNFRSF1A*	0.60	7.41E-10	*TSPO*	*TNFRSF1A*	0.66	6.52E-20
				*TSPO*	*MYD88*	0.66	6.98E-20
				*TLR2*	*CD68*	0.66	1.16E-19
				*AIF1*	*CX3CR1*	0.66	2.20E-19
				*TNFRSF1A*	*MYD88*	0.65	5.94E-19
				*AIF1*	*TLR2*	0.65	1.12E-18
				*TSPO*	*IL1R1*	0.64	2.63E-18
				*TSPO*	*GFAP*	0.63	1.26E-17
				*MAP2*	*SNCA*	0.63	1.64E-17
				*TLR2*	*MYD88*	0.62	3.05E-17
				*TSPO*	*MAP2*	−0.60	7.67E-16
				*MYD88*	*CASP1*	0.60	1.10E-15
				*IL1R1*	*MYD88*	0.60	1.16E-15
				*TSPO*	*SNCA*	−0.60	1.41E-15

In p-values, term E-number = ×10^−number^.

### Gene Expression and Chromosome Proximity

Four pairs of genes have the same band number on a chromosome: *PLA2G4A* and *PTGS2* (1q25); *IL1β* and *IL1RN* (2q14); *CX3CR1* and *MOBP* (3p21.3); and *AIF1* and *MAPK14* (6p21.3) (cf. Table S1). At p<0.001, expression levels of *PLA2G4A* and *PTGS2* were correlated positively during both Development and Aging, while expression levels of *IL1β* and *IL1RN* were correlated positively during Development only. Expression levels of the other two gene pairs were not correlated significantly in either period.

## Discussion

We used the BrainCloud database for human prefrontal cortex [48] to examine age variations in mRNA levels of 39 genes reported to be involved in pathways of neuroinflammation, cytokine signaling, arachidonic acid metabolism, neuronal and myelin integrity, synaptic function, neurotrophic action, and related processes. We divided the postnatal lifespan into Development (0–21 years) and Aging (22 to 78 years) intervals, on the basis of reported distinct functional and structural brain changes in these periods [4], [5], [47], [49], [72].

Confirming this division, expression patterns and age correlations of many of the chosen genes frequently differed significantly between the two intervals. Genes with higher expression during Aging include *TSPO*, associated with microglial activation and cholesterol transport [73], [74]; *GFAP*, associated with glial activation [71]; *TRAF6*, associated with TNFα signaling; *MOBP*, associated with myelin integrity; and *NFKB1*, coding for NF-κB, a major transcription factor of inflammatory genes involved in innate immunity [25]. More genes were expressed at lower levels during Aging than Development, reflecting a reduced intensity of early developmental events during Aging. These genes are related to synaptic integrity, neuronal growth, neurotrophic, glial changes and other development modifications (*CX3CR1*, *CX3CL1*, *PTGS2*, *BDNF*, *CASP1*, *CD68*, *AIF1*, *MYD88*, *NGF*, *PDGFA*, *DBN1*, *IL1B*, *SYP*, and *BACE1*).

Neurodevelopment is influenced largely by programmed transcriptional changes involving neuronal, glial and synaptic integrity, and myelination, whereas after 21 years of age, gene expression reaches a homeostatic state that depends more on factors such as health status, environment, and nutrition [48], [57], [75], [76]. Late senescence becomes a risk factor for neurodegenerative diseases such as Alzheimer's and Parkinson's disease, when the brain shows increased inflammatory, apoptotic, and arachidonic cascade markers, but reduced neurotrophic and synaptic markers [9], [13], [15], [77], [78], [79]. Our data in the Aging interval indicate many significant positive age correlations in expression of genes in the former category (*GFAP, TSPO, TRAF6, NFKB1, TLR4, IL1R1*) but decreased correlations for genes in the latter category (*BDNF, SYP, SNCA, NGF*) (Figure 2). These findings support the proposition that aging increases vulnerability to neurodegenerative disease and is a priming factor for it [9], [13], [15], [26], [27], [28], [29], [30], [31], [77], [78], [79].

The significant age changes in mRNA levels during the Aging interval do not always correspond to reported protein changes. TNFα and IFI-16 protein levels were reported to increase between 26 to 106 years in postmortem human brain [35], whereas we did not find age increases in *TNFRSF1A* or *IFI16* expression in the Aging interval (Figure 2), or on average between Aging and Development (Table 1). On the other hand, and consistent with our expression changes, positive age correlations between 42 and 70 years were reported in brain mRNA and protein levels of GFAP, IL-1β, iNOS, NF-κB p50, cPLA_2_ IVA and COX-2, while levels of BDNF and SYP declined in this period [34]. Some of the changes correlated with promoter hypermethylation of *BDNF* and cyclic AMP responsive element binding protein (*CREB*), and hypomethylation of Bcl-2 associated X protein (*BAX*), suggesting epigenetic influence [80]. Another study also reported a decrease in BDNF protein during the Aging interval [81], while another reported decreased protein levels of DBN1, GAP-43, and SYN [9]. In general agreement, we found significant age increases in expression of *GFAP*, *IL1R1*, and *NFKB1*, and reductions in expression of *BDNF* and *SYP* (Table 1).

Age correlations often differed between the Development and Aging intervals, indicating different roles for the gene products over the lifespan (Figures 2 and 3, Table 1). Expression of *SNCA* increased during Development but decreased during Aging; expression of *PLA2G4A*, *PTGS1*, *TRAF6*, *TLR4*, *APP*, and *IL1R1* increased and expression of *DBN1*, *MYD88*, *RELA*, *PDGF*, and *IFI16* decreased during Development alone. Expression of *MOBP* increased during both intervals, consistent with continued myelination into the fourth decade in frontal cortex [5], as did expression of *NFKB1*, while expression of *NGF* decreased during both periods, suggesting reduced neuroplasticity [82]. The NFκB1 system can be stimulated by a number of cell surface receptors (Figure 1), as well as by oxidative stress, hypoxia, and genotoxic stress [25]. In non-stimulated cells, NF-κB complexes are bound in cytoplasm to inhibitory I-kappa-B (IκB) proteins. Stimulation phosphorylates IκB proteins, which are ubiquitinated and broken down, allowing the NF-κB complex to enter the nucleus and activate transcription of multiple genes, particularly related to inflammatory cascades [25].

In the first six months of life, a number of genes showed non-linear expression changes that later reach a plateau (Figure 4). In this same period, neuronal density in layers 2–3 of human frontal cortex falls by 80% [3]. However, dendritic spine densities at different levels of prefrontal cortical pyramidal neurons rise from birth to about 5–10 years of age, and then decline [56], [83]. Metabolic changes also occur, as the brain shifts from using ketone bodies to glucose in the first months of life [6]. Expression of *GAP43* and *DBN1*, coding for presynaptic GAP43 and postsynaptic dendritic spine drebrin, decreased nonlinearly. As dendritic spine density increases in the first 5–10 years (see above), *DBN1* and *GAP43* likely change in this period. Expression increased for *PLA2G4A* and *PTGS2* coding for postsynaptic functionally-coupled cPLA_2_ Type IVA and COX-2 [84], [85], [86] suggesting a growing role for arachidonic acid signaling in neurotransmission at dendritic spines [87], [88].

Increases in *TLR4* and *IL1R1* in the first years of life may reflect increased receptivity of microglia, on which these receptors are located [89], as microglia participate postnatally in synaptic pruning and apoptosis [38], [39], [40], [41]. In this regard, higher (2-fold) levels of expression of *CX3CR1* (chemokine (C-X3-C motif) receptor 1) and of its ligand fractalkine (*CX3CL1*) during Development than Aging highlight the importance their protein products in early neuronal-glial interactions. *CX3CR1* is expressed exclusively by microglia in brain [90], whereas *CX3CL1* is highly expressed in neurons. Knocking out *CX3CR1* reduced neuron loss [91] and amyloid-beta deposition [92] in Alzheimer's disease mouse models, and interfered with formation of thalamocortical synapses during development, when fractalkine is overexpressed.

Gene expression clusters in the heat map matrices of Figures 5a and 5b identify genes having high intercorrelated expression patterns as the brain ages. The clusters were similar in the Development and Aging intervals. Thus, comparable transcriptional regulatory networks operate throughout the life span, but underlie different phenotypic processes during Aging compared to Development. Gene products within Cluster 1 of both Development and Aging (*GAP43*, *SYN*, *BDNF*, *NOS2*, *SNCA*) are involved in synaptic signaling and integrity, cellular stress, and neurogenesis. Gene products in Cluster 2 of both periods (*BACE1*, *CX3CR1*, *PTGS1*, and *PLA2G4A*) are involved in the arachidonic acid cascade, protease activity, APP processing, and inflammatory processing. Gene products in Cluster 3 of both groups (*TSPO*, *TNFRSF1A*, *IL1R1*, *MYD88*, *CD68*, *AIF1*, *CASP1*, and *TLR2*) are involved in microglial, inflammasome, NF-κB signaling, and various neuroinflammatory responses.

Pairwise correlations, whether positive or negative, are more frequently significant during the Aging than Development interval (Figures S1a and S1b, Table 2). This may reflect our selective choice of genes, but if confirmed would suggest a more stable state of synchronized gene expression in the Aging than Development interval. This is likely since genome-wide promoter DNA methylation of CpG dinucleotides in human prefrontal cortex changes less in adulthood than early childhood [57]. Mean cortical global methylation is increased in the Aging interval, which may reflect some gene silencing [57], but global methylation is higher in late senescence [34].

Since the subjects were considered to be healthy, some presumably deleterious expression changes in the Aging interval had not progressed sufficiently to produce noticeable functional deficits, although they may have increased vulnerability to stress and other disease factors. Upregulated translator protein (*TSPO*) has been imaged using positron emission tomography in patients with mild cognitive impairment (MCI) and Alzheimer's disease [73], [93], [94]. Thus, the higher *TSPO* expression in Aging than Development is consistent with late-stage “inflamm-aging” in presumably healthy subjects (Table 1) [26]. In support, expression levels of *TRAF6*, *NFKB1*, *TLR4*, and *IL1R1*, important in the initial inflammatory response involving NF-κB (Figure 1), and of *GFAP*, were upregulated in the Aging interval. Nevertheless, not all gene markers of inflammation or microglial/astrocytic activation were upregulated. Expression levels of inflammation-related genes *CASP1*, *PTGS2*, *MYD88*, and *IL1B*, and of microglia genes *CD68* and *AIF1* (also known as IBA1), were reduced in the Aging group.

Our age correlations identified unexpected relations between different genes. Highly significant pairwise correlations were found between expression levels of *APP* and *MAP2*, of *AIF1* (IBA1) and *CD68*, and of *IL1R1* and *TNFRSF1A*. *APP*, a component in Alzheimer's disease pathogenesis, normally helps to maintain functional synapses [95]. Microtubule-associated protein 2 (MAP2) is found in post-synaptic dendrites and is functionally similar to tau protein, whose abnormal phosphorylation is another key component in Alzheimer's disease. *AIF1* (IBA1), expressed in macrophages and microglia, and *CD68*, expressed in macrophages and monocytes, contribute to the inflammatory response in brain. Also, *IL1R1* and the TNFα receptor (*TNFRSF1A*) are important in initial signaling in inflammation (Figure 1). Among the other highly correlated genes, *CASP1* is involved in inflammasome formation [96], and *TSPO* is upregulated during neuroinflammation. Disturbed α-synuclein (*SNCA*) and APP occurs in Parkinson's disease and Alzheimer's disease respectively, and expression of *SNCA* and *APP* was very highly correlated in Development (r = 0.67) and in Aging (r = 0.56) (Figure S1). The Aging interval had a greater number of significant correlations between genes, and many significant correlations occurred consistently in Development and Aging. The correlated expression of genes in the canonical pathway of *NF-κB* (*NFKB1, MYD88, TLR4, IL1R1, TRAF6*) also showed a highly integrated network of genes with similar expression patterns with age [25].

Significant correlations in gene expression that corresponded to chromosome proximity (Table S1) for *PLA2G4A* (cPLA_2_ IVA) and *PTGS2* (COX-2) at locus 1q25, and for *IL1R1*, *IL1B*, and *IL1RN* at locus 2q14 indicate robust co-regulatory elements and possible coevolution [47], [97], [98], [99]. The IL-1 receptor type 1 (*IL1R1*), a key cytokine receptor in innate immunity, interacts with *IL1B* and the IL-1 receptor antagonist (*IL1RN*) in a complex cascade (Figure 1) [36]. During Development, expression of *IL1B* and *IL1RN* exhibited a strong positive correlation (p<0.001), but their expression was not correlated significantly during Aging. Increased IL-1R signaling has been implicated in bipolar disorder, Alzheimer's disease, and HIV-1 encephalopathy [100], [101], [102]. Our changes during Development and Aging are consistent with neuroregulatory as well as neuroinflammatory functions involving the NF-κB transcription system.

Some of the expression changes with senescence in this study correspond to changes reported in postmortem frontal cortex from Alzheimer's disease, bipolar disorder, and schizophrenia patients, compared to age-matched non-pathological cortex. Alzheimer's disease cortex showed significantly increased mRNA and protein levels of IL-1β, TNFα, GFAP, CD11b, cPLA_2_ IVA sPLA_2_ IIA COX-1 and COX-2 [13], [103], [104], but decreased levels of pre-synaptic synaptophysin (SYP) and post-synaptic drebrin (DBN1) [9], [13]. In bipolar disorder, protein and mRNA levels of neuroinflammatory markers (IL-1β, IL-1R, MYD88, NF-kB1) and of activated microglia and astroglial markers (GFAP, NOS2, c-Fos, and CD11b) also were significantly higher than in control cortex [69], [105]. These changes were accompanied by reduced expression of anti-apoptotic factors B cell lymphoma (Bcl)-2, BDNF, SYN, and DBN1, but increased expression of pro-apoptotic Bax, BAD, and active caspase (CASP)-3 and CASP-9 [15]. Ca^2+^-dependent cPLA_2_ IVA, secretory sPLA_2_ IIA and COX-2 also were overexpressed [70]. Similar changes were noted in schizophrenic frontal cortex [71].

This study has several limitations. Selection criteria for the microarray gene probes were a way to biologically standardize all probes by their protein-coding regions. There are other criteria of selection; however, we found that taking mean expression data of all probes for a given gene was not a good representation of gene's expression (data not shown), as expression levels differed between probes of the same gene.

BrainCloud contains expression data selective to the prefrontal cortex [48]. The prefrontal cortex shows prolonged development and preliminary degradation associated with aging earlier than other brain regions [5], [106], [107]. Other studies have found differential gene expressed based on cell type, such as the Allen Brain Atlases and the Loerch study; however, BrainCloud did not differentiate between cell types [108], [109], [110]. Favorably, BrainCloud has a large number of samples (n = 269) compared with other aging databases with time points through a lifespan. For further discussion on BrainCloud and its application, see publications [47], [48].

In the future, it would be of interest to consider mechanisms underlying the age-related expression changes. These may involve histone acetylation and methylation, transcription factors, miRNAs, DNA sequences of cis-elements (transcription factor binding sites), all of which can influence mRNA expression [57], [111], [112], [113]. In this regard, many genes whose expression decreases with age appear to have higher promoter GC content than other genes [49], suggesting differences in methylation state, and human brain aging is associated with changes in global methylation [34], [57]. As we considered only two transcription factors in this study, *TFAP2A* (AP-2) and *NFKB1* (NF-κB), future aging studies may consider more.

## Supporting Information

Figure S1 **Matrices showing Pearson correlation coefficients between expression levels of individual gene pairs during Development (A) and Aging (B).** Green highlights coefficients that are significant at p<0.0001. Hierarchy in gene order corresponds to hierarchy in Figures 5A and 5B.
(XLSX)

Table S1 **Selected genes, chromosomal locations, protein description, and major reported functions.** Based on the literature, genes whose protein products participate in major functions may be categorized as follows: (1) Synaptic function, *SYP, DBN1, SNCA*; (2) Growth and maintenance, *BDNF, NGF, GAP43, PDGFA*; (3) Myelin integrity, *MOBP*; (4) Neuroinflammation: (a) Microglial activation, *CD68, TSPO, TLR4, TLR2, NOS2, AIF1*; (b) Cytokine and chemokine processes, *CX3CR1, CX3CL1, IFI16, IL1B, IL1R1, IL1RN, IL1RAP, IL6, TNFRSF1A, TRAF6, TRAP1, MAPK14, MYD88*; (c) Glial activation, *GFAP*; (d) Apoptosis, *CASP1*; (5) Arachidonic acid cascade, *PLA2G4A, PLA2G10, PTGS1, PTGS2*; (5) Amyloid membrane processing, *APP, BACE*; (6) Microtubules, *MAP2*; (7) Transcription factors, *NFKB1, TFAP2A* [61], [62], [64], [114].
(DOCX)

## References

[1] de Graaf-Peters VB, Hadders-Algra M (2006) Ontogeny of the human central nervous system: what is happening when? Early human development 82: 257–266. 16360292 10.1016/j.earlhumdev.2005.10.013

[2] Hedden T, Gabrieli JDE (2004) Insights into the ageing mind: a view from cognitive neuroscience. Nature reviews Neuroscience 5: 87–96. 14735112 10.1038/nrn1323

[3] Huttenlocher PR (1984) Synapse elimination and plasticity in developing human cerebral cortex. American journal of mental deficiency 88: 488–496. 6731486

[4] Huttenlocher PR (1990) Morphometric study of human cerebral cortex development. Neuropsychologia 28: 517–527. 2203993 10.1016/0028-3932(90)90031-i

[5] Yakovlev PI, Lecours AR (1967) The myelogenetic cycles of regional maturation of the brain. In: Minkowski A, editor. Regional Development of the Brain in Early Life. Philadelphia: F. A. Davis. pp. 3–70.

[6] Chugani HT, Hovda DA, Villablanca JR, Phelps ME, Xu WF (1991) Metabolic maturation of the brain: a study of local cerebral glucose utilization in the developing cat. Journal of cerebral blood flow and metabolism: official journal of the International Society of Cerebral Blood Flow and Metabolism 11: 35–47. 10.1038/jcbfm.1991.4 1984003

[7] Sowell ER, Thompson PM, Toga AW (2004) Mapping changes in the human cortex throughout the span of life. The Neuroscientist: a review journal bringing neurobiology, neurology and psychiatry 10: 372–392. 10.1177/1073858404263960 15271264

[8] Rapoport SI (1990) Integrated phylogeny of the primate brain, with special reference to humans and their diseases. Brain research Brain research reviews 15: 267–294. 2289087 10.1016/0165-0173(90)90004-8

[9] Hatanpää K, Hatanpää K, Isaacs KR, Isaacs KR, Shirao T, et al (1999) Loss of proteins regulating synaptic plasticity in normal aging of the human brain and in Alzheimer disease. Journal of neuropathology and experimental neurology 58: 637–643. 10374754 10.1097/00005072-199906000-00008

[10] Azari NP, Rapoport SI, Salerno JA, Grady CL, Gonzalez-Aviles A, et al (1992) Interregional correlations of resting cerebral glucose metabolism in old and young women. Brain research 589: 279–290. 1393596 10.1016/0006-8993(92)91288-p

[11] Creasey H, Rapoport SI (1985) The aging human brain. Annals of neurology 17: 2–10. 3885841 10.1002/ana.410170103

[12] Koss E, Haxby JV, DeCarli CS, Schapiro MB, Friedland RP, et al (1991) Patterns of performance preservation and loss in healthy aging. Dev Neuropsychol 7: 99–113.

[13] Rao JS, Rapoport SI, Kim H-W (2011) Altered neuroinflammatory, arachidonic acid cascade and synaptic markers in postmortem Alzheimer's disease brain. Translational psychiatry 1: e31. 22832605 10.1038/tp.2011.27 PMC3309508

[14] Rao JS, Kellom M, Kim H-W, Rapoport SI, Reese EA (2012) Neuroinflammation and synaptic loss. Neurochemical research 37: 903–910. 22311128 10.1007/s11064-012-0708-2 PMC3478877

[15] Kim H-W, Rapoport SI, Rao JS (2010) Altered expression of apoptotic factors and synaptic markers in postmortem brain from bipolar disorder patients. Neurobiology of disease 37: 596–603. 19945534 10.1016/j.nbd.2009.11.010 PMC2823851

[16] Wingo AP, Harvey PD, Baldessarini RJ (2009) Neurocognitive impairment in bipolar disorder patients: functional implications. Bipolar disorders 11: 113–125. 19267694 10.1111/j.1399-5618.2009.00665.x

[17] McGeer PL, McGeer EG (2004) Inflammation and the Degenerative Diseases of Aging. Annals of the New York Academy of Sciences 1035: 104–116. 15681803 10.1196/annals.1332.007

[18] Perry VH, Bolton SJ, Anthony DC, Betmouni S (1998) The contribution of inflammation to acute and chronic neurodegeneration. Research in immunology 149: 721–725. 9851531 10.1016/s0923-2494(99)80046-7

[19] Kraft AD, Kraft AD, Harry GJ, Harry GJ (2011) Features of microglia and neuroinflammation relevant to environmental exposure and neurotoxicity. International journal of environmental research and public health 8: 2980–3018. 21845170 10.3390/ijerph8072980 PMC3155341

[20] Kumar A, Loane DJ (2012) Neuroinflammation after traumatic brain injury: opportunities for therapeutic intervention. Brain, behavior, and immunity 26: 1191–1201. 10.1016/j.bbi.2012.06.008 22728326

[21] Choi SH, Aid S, Bosetti F (2009) The distinct roles of cyclooxygenase-1 and -2 in neuroinflammation: implications for translational research. Trends in pharmacological sciences 30: 174–181. 19269697 10.1016/j.tips.2009.01.002 PMC3379810

[22] Hoeck WG, Hoeck WG, Ramesha CS, Ramesha CS, Chang DJ, et al (1993) Cytoplasmic phospholipase A2 activity and gene expression are stimulated by tumor necrosis factor: dexamethasone blocks the induced synthesis. Proceedings of the National Academy of Sciences of the United States of America 90: 4475–4479. 8506288 10.1073/pnas.90.10.4475 PMC46534

[23] Spriggs DR, Sherman ML, Imamura K, Mohri M, Rodriguez C, et al (1990) Phospholipase A2 activation and autoinduction of tumor necrosis factor gene expression by tumor necrosis factor. Cancer research 50: 7101–7107. 2121330

[24] Bauer MK, Bauer MK, Lieb K, Lieb K, Schulze-Osthoff K, et al (1997) Expression and regulation of cyclooxygenase-2 in rat microglia. European journal of biochemistry/FEBS 243: 726–731. 10.1111/j.1432-1033.1997.00726.x 9057838

[25] Salminen A, Huuskonen J, Ojala J, Kauppinen A, Kaarniranta K, et al (2008) Activation of innate immunity system during aging: NF-kB signaling is the molecular culprit of inflamm-aging. Ageing research reviews 7: 83–105. 17964225 10.1016/j.arr.2007.09.002

[26] Franceschi C, Capri M, Monti D, Giunta S, Olivieri F, et al (2007) Inflammaging and anti-inflammaging: a systemic perspective on aging and longevity emerged from studies in humans. Mechanisms of ageing and development 128: 92–105. 17116321 10.1016/j.mad.2006.11.016

[27] VanGuilder HD, Bixler GV, Brucklacher RM (2011) Concurrent hippocampal induction of MHC II pathway components and glial activation with advanced aging is not correlated with cognitive impairment. J … 10.1186/1742-2094-8-138 PMC3216278 21989322

[28] Dilger RN, Johnson RW (2008) Aging, microglial cell priming, and the discordant central inflammatory response to signals from the peripheral immune system. Journal of leukocyte biology 84: 932–939. 18495785 10.1189/jlb.0208108 PMC2538600

[29] Letiembre M, Hao W, Liu Y, Walter S, Mihaljevic I (2007) Innate immune receptor expression in normal brain aging. Neuroscience 10.1016/j.neuroscience.2007.01.004 17293054

[30] Griffin R, Nally R, Nolan Y, McCartney Y, Linden J, et al (2006) The age-related attenuation in long-term potentiation is associated with microglial activation. Journal of neurochemistry 99: 1263–1272. 16981890 10.1111/j.1471-4159.2006.04165.x

[31] Goldstein BI, Kemp DE, Soczynska JK, McIntyre RS (2009) Inflammation and the phenomenology, pathophysiology, comorbidity, and treatment of bipolar disorder: a systematic review of the literature. The Journal of clinical psychiatry 70: 1078–1090. 19497250 10.4088/JCP.08r04505

[32] Frank MG, Barrientos RM, Biedenkapp JC, Rudy JW (2006) mRNA up-regulation of MHC II and pivotal pro-inflammatory genes in normal brain aging. Neurobiology of … 10.1016/j.neurobiolaging.2005.03.013 15890435

[33] Sheffield LG, Berman N (1998) Microglial expression of MHC class II increases in normal aging of nonhuman primates. Neurobiology of Aging 10.1016/s0197-4580(97)00168-1 9562503

[34] Keleshian VL, Modi HR, Rapoport SI, Rao JS (2013) Aging is associated with altered inflammatory, arachidonic acid cascade, and synaptic markers, influenced by epigenetic modifications, in the human frontal cortex. Journal of Neurochemistry 125: 63–73. 23336521 10.1111/jnc.12153 PMC3606672

[35] Lu T, Pan Y, Kao S-Y, Li C, Kohane I, et al (2004) Gene regulation and DNA damage in the ageing human brain. Nature 429: 883–891. 15190254 10.1038/nature02661

[36] Dinarello CA (2002) The IL-1 family and inflammatory diseases. Clin Exp Rheumatol 20: S1–13. 14989423

[37] Buka SL, Tsuang MT, Torrey EF, Klebanoff MA, Wagner RL, et al (2001) Maternal cytokine levels during pregnancy and adult psychosis. Brain, behavior, and immunity 15: 411–420. 10.1006/brbi.2001.0644 11782107

[38] Garden GA, Moller T (2006) Microglia biology in health and disease. Journal of neuroimmune pharmacology: the official journal of the Society on NeuroImmune Pharmacology 1: 127–137. 18040779 10.1007/s11481-006-9015-5

[39] Kim SU, de Vellis J (2005) Microglia in health and disease. Journal of neuroscience research 81: 302–313. 15954124 10.1002/jnr.20562

[40] Deverman BE, Patterson PH (2009) Cytokines and CNS development. Neuron 64: 61–78. 19840550 10.1016/j.neuron.2009.09.002

[41] Kaneko M, Stellwagen D, Malenka RC, Stryker MP (2008) Tumor necrosis factor-alpha mediates one component of competitive, experience-dependent plasticity in developing visual cortex. Neuron 58: 673–680. 18549780 10.1016/j.neuron.2008.04.023 PMC2884387

[42] Johnstone DM, Graham RM, Trinder D, Riveros C, Olynyk JK, et al (2012) Changes in brain transcripts related to Alzheimer's disease in a model of HFE hemochromatosis are not consistent with increased Alzheimer's disease risk. Journal of Alzheimer's disease: JAD 30: 791–803. 22466002 10.3233/JAD-2012-112183

[43] Guet CC, Elowitz MB, Hsing W, Leibler S (2002) Combinatorial synthesis of genetic networks. Science 296: 1466–1470. 12029133 10.1126/science.1067407

[44] Crespo I, Roomp K, Jurkowski W, Kitano H, del Sol A (2012) Gene regulatory network analysis supports inflammation as a key neurodegeneration process in prion disease. BMC systems biology 6: 132. 23068602 10.1186/1752-0509-6-132 PMC3607922

[45] Dieci G, Fermi B, Bosio MC (2014) Investigating transcription reinitiation through in vitro approaches. Transcription 5. 10.4161/trns.27704 PMC4214232 25764113

[46] Hashimshony T, Yanai I (2010) Revealing developmental networks by comparative transcriptomics. Transcription 1: 154–158. 21326891 10.4161/trns.1.3.13190 PMC3023577

[47] Ryan VH, Primiani CT, Rao JS, Ahn K, Rapoport SI, et al (2014) Coordination of Gene Expression of Arachidonic and Docosahexaenoic Acid Cascade Enzymes during Human Brain Development and Aging. PLoS ONE 9 6: e100858 doi:10.1371/journal.pone.0100858 24963629 10.1371/journal.pone.0100858 PMC4070994

[48] Colantuoni C, Lipska BK, Ye T, Hyde TM, Tao R, et al (2011) Temporal dynamics and genetic control of transcription in the human prefrontal cortex. Nature 478: 519–523. 22031444 10.1038/nature10524 PMC3510670

[49] Somel M, Guo S, Fu N, Yan Z, Hu HY, et al (2010) MicroRNA, mRNA, and protein expression link development and aging in human and macaque brain. Genome research 20: 1207–1218. 20647238 10.1101/gr.106849.110 PMC2928499

[50] Rodwell GEJ, Sonu R, Zahn JM, Lund J, Wilhelmy J, et al (2004) A transcriptional profile of aging in the human kidney. PLoS biology 2: e427. 15562319 10.1371/journal.pbio.0020427 PMC532391

[51] Norden DM, Godbout JP (2013) Review: Microglia of the aged brain: primed to be activated and resistant to regulation. Neuropathology and Applied Neurobiology 39: 19–34. 23039106 10.1111/j.1365-2990.2012.01306.x PMC3553257

[52] Nichols NR, Day JR, Laping NJ, Johnson SA, Finch CE (1993) GFAP mRNA increases with age in rat and human brain. Neurobiology of Aging 14: 421–429. 8247224 10.1016/0197-4580(93)90100-p

[53] Iwama H (2013) Coordinated networks of microRNAs and transcription factors with evolutionary perspectives. Advances in experimental medicine and biology 774: 169–187. 23377974 10.1007/978-94-007-5590-1_10

[54] Jo YH, Chua S Jr (2009) Transcription factors in the development of medial hypothalamic structures. American journal of physiology Endocrinology and metabolism 297: E563–567. 19383874 10.1152/ajpendo.00064.2009 PMC2739694

[55] Uffenbeck SR, Krebs JE (2006) The role of chromatin structure in regulating stress-induced transcription in Saccharomyces cerevisiae. Biochemistry and cell biology = Biochimie et biologie cellulaire 84: 477–489. 16936821 10.1139/o06-079

[56] Goyal MS, Raichle ME (2013) Gene expression-based modeling of human cortical synaptic density. Proceedings of the National Academy of Sciences of the United States of America 110: 6571–6576. 23576754 10.1073/pnas.1303453110 PMC3631628

[57] Numata S, Ye T, Hyde TM, Guitart-Navarro X, Tao R, et al (2012) DNA methylation signatures in development and aging of the human prefrontal cortex. American journal of human genetics 90: 260–272. 22305529 10.1016/j.ajhg.2011.12.020 PMC3276664

[58] Kang HJ, Kawasawa YI, Cheng F, Zhu Y, Xu X, et al (2011) Spatio-temporal transcriptome of the human brain. Nature 478: 483–489. 22031440 10.1038/nature10523 PMC3566780

[59] Benjamini Y, Y H (1995) Controlling the false discovery rate: A practical and powerful approach to multiple testing. J Royal Statistical Soc Ser B 57: 289–300.

[60] Johnson WE, Li C, Rabinovic A (2007) Adjusting batch effects in microarray expression data using empirical Bayes methods. Biostatistics (Oxford, England) 8: 118–127. 10.1093/biostatistics/kxj037 16632515

[61] Boeckmann B, Blatter MC, Famiglietti L, Hinz U, Lane L, et al (2005) Protein variety and functional diversity: Swiss-Prot annotation in its biological context. C R Biol 328: 882–899. 16286078 10.1016/j.crvi.2005.06.001

[62] Pruitt KD, Tatusova T, Brown GR, Maglott DR (2012) NCBI Reference Sequences (RefSeq): current status, new features and genome annotation policy. Nucleic Acids Res 40: D130–135. 22121212 10.1093/nar/gkr1079 PMC3245008

[63] Pruitt KD, Tatusova T, Klimke W, Maglott DR (2009) NCBI Reference Sequences: current status, policy and new initiatives. Nucleic Acids Res 37: D32–36. 18927115 10.1093/nar/gkn721 PMC2686572

[64] Rebhan M, Chalifa-Caspi V, Prilusky J, Lancet D (1997) GeneCards: integrating information about genes, proteins and diseases. Trends in Genetics 13: 163. 9097728 10.1016/s0168-9525(97)01103-7

[65] De Trez C, Ware CF (2008) The TNF receptor and Ig superfamily members form an integrated signaling circuit controlling dendritic cell homeostasis. Cytokine & growth factor reviews 19: 277–284. 18511331 10.1016/j.cytogfr.2008.04.013 PMC2581770

[66] Troutman TD, Bazan JF, Pasare C (2012) Toll-like receptors, signaling adapters and regulation of the pro-inflammatory response by PI3K. Cell cycle 11: 3559–3567. 22895011 10.4161/cc.21572 PMC3478307

[67] Lehnardt S, Massillon L, Follett P, Jensen FE, Ratan R, et al (2003) Activation of innate immunity in the CNS triggers neurodegeneration through a Toll-like receptor 4-dependent pathway. Proc Natl Acad Sci U S A 100: 8514–8519. 12824464 10.1073/pnas.1432609100 PMC166260

[68] Basu A, Krady JK, Levison SW (2004) Interleukin-1: a master regulator of neuroinflammation. Journal of neuroscience research 78: 151–156. 15378607 10.1002/jnr.20266

[69] Rao JS, Harry GJ, Rapoport SI, Kim HW (2010) Increased excitotoxicity and neuroinflammatory markers in postmortem frontal cortex from bipolar disorder patients. Molecular psychiatry 15: 384–392. 19488045 10.1038/mp.2009.47 PMC2844920

[70] Kim HW, Rapoport SI, Rao JS (2011) Altered arachidonic acid cascade enzymes in postmortem brain from bipolar disorder patients. Molecular psychiatry 16: 419–428. 20038946 10.1038/mp.2009.137 PMC3190400

[71] Rao JS, Kim HW, Harry GJ, Rapoport SI, Reese EA (2013) Increased neuroinflammatory and arachidonic acid cascade markers, and reduced synaptic proteins, in the postmortem frontal cortex from schizophrenia patients. Schizophrenia research 147: 24–31. 23566496 10.1016/j.schres.2013.02.017 PMC3812915

[72] Shaw P, Eckstrand K, Sharp W, Blumenthal J, Lerch JP, et al (2007) Attention-deficit/hyperactivity disorder is characterized by a delay in cortical maturation. Proceedings of the National Academy of Sciences of the United States of America 104: 19649–19654. 18024590 10.1073/pnas.0707741104 PMC2148343

[73] Hommet C, Mondon K, Camus V, Ribeiro MJ, Beaufils E, et al (2013) Neuroinflammation and β Amyloid Deposition in Alzheimer's Disease: In vivo Quantification with Molecular Imaging. Dementia and geriatric cognitive disorders 37: 1–18. 24107621 10.1159/000354363

[74] Taylor JM, Allen AM, Graham A (2014) Targeting mitochondrial 18 kDa Translocator protein (TSPO) regulates macrophage cholesterol efflux and lipid phenotype. Clinical science 10.1042/CS20140047 24814875

[75] Lipska BK, Weinberger DR (1995) Genetic variation in vulnerability to the behavioral effects of neonatal hippocampal damage in rats. Proc Natl Acad Sci U S A 92: 8906–8910. 7568041 10.1073/pnas.92.19.8906 PMC41076

[76] Shankar SK (2010) Biology of aging brain. Indian journal of pathology & microbiology 53: 595–604. 21045377 10.4103/0377-4929.71995

[77] Glass CK, Saijo K, Winner B, Winner B, Marchetto MC, et al (2010) Mechanisms underlying inflammation in neurodegeneration. Cell 140: 918–934. 20303880 10.1016/j.cell.2010.02.016 PMC2873093

[78] Allan SM, Rothwell NJ (2003) Inflammation in central nervous system injury. Philosophical transactions of the Royal Society of London Series B, Biological sciences 358: 1669–1677. 14561325 10.1098/rstb.2003.1358 PMC1693261

[79] Caldeira GL, Ferreira IL, Rego AC (2013) Impaired transcription in Alzheimer's disease: key role in mitochondrial dysfunction and oxidative stress. Journal of Alzheimer's disease: JAD 34: 115–131. 23364141 10.3233/JAD-121444

[80] Lister R, Mukamel EA, Nery JR, Urich M, Puddifoot CA, et al (2013) Global epigenomic reconfiguration during mammalian brain development. Science 341: 1237905. 23828890 10.1126/science.1237905 PMC3785061

[81] Rao J, Chiappelli J, Kochunov P, Rapoport SI, Hong LE (In preparation) Age Changes in Brain Derived Neurotrophic Factor in Postmortem Gray and White Matter from Schizophrenia and Control Subjects.

[82] Terry AV Jr, Kutiyanawalla A, Pillai A (2011) Age-dependent alterations in nerve growth factor (NGF)-related proteins, sortilin, and learning and memory in rats. Physiology & behavior 102: 149–157. 21059364 10.1016/j.physbeh.2010.11.005 PMC3010498

[83] Petanjek Z, Judas M, Simic G, Rasin MR, Uylings HB, et al (2011) Extraordinary neoteny of synaptic spines in the human prefrontal cortex. Proceedings of the National Academy of Sciences of the United States of America 108: 13281–13286. 21788513 10.1073/pnas.1105108108 PMC3156171

[84] Kaufmann WE, Worley PF, Pegg J, Bremer M, Isakson P (1996) COX-2, a synaptically induced enzyme, is expressed by excitatory neurons at postsynaptic sites in rat cerebral cortex. Proc Natl Acad Sci U S A 93: 2317–2321. 8637870 10.1073/pnas.93.6.2317 PMC39793

[85] Ong WY, Yeo JF, Ling SF, Farooqui AA (2005) Distribution of calcium-independent phospholipase A2 (iPLA 2) in monkey brain. J Neurocytol 34: 447–458. 16902765 10.1007/s11068-006-8730-4

[86] Murakami M, Kambe T, Shimbara S, Kudo I (1999) Functional coupling between various phospholipase A2s and cyclooxygenases in immediate and delayed prostanoid biosynthetic pathways. J Biol Chem 274: 3103–3105. 9915849 10.1074/jbc.274.5.3103

[87] Rapoport SI (2008) Arachidonic acid and the brain. The Journal of nutrition 138: 2515–2520. 19022981 10.1093/jn/138.12.2515 PMC3415870

[88] Ramadan E, Rosa AO, Chang L, Chen M, Rapoport SI, et al (2010) Extracellular-derived calcium does not initiate in vivo neurotransmission involving docosahexaenoic acid. J Lipid Res 51: 2334–2340. 20388940 10.1194/jlr.M006262 PMC2903827

[89] Harry GJ (2013) Microglia during development and aging. Pharmacology & therapeutics 139: 313–326. 23644076 10.1016/j.pharmthera.2013.04.013 PMC3737416

[90] Cardona AE, Pioro EP, Sasse ME, Kostenko V, Cardona SM, et al (2006) Control of microglial neurotoxicity by the fractalkine receptor. Nature neuroscience 9: 917–924. 16732273 10.1038/nn1715

[91] Fuhrmann M, Bittner T, Jung CKE, Burgold S, Page RM, et al (2010) Microglial Cx3cr1 knockout prevents neuron loss in a mouse model of Alzheimer's disease. Nature neuroscience 13: 411–413. 20305648 10.1038/nn.2511 PMC4072212

[92] Lee S, Varvel NH, Konerth ME, Xu G, Cardona AE, et al (2010) CX3CR1 deficiency alters microglial activation and reduces beta-amyloid deposition in two Alzheimer's disease mouse models. The American journal of pathology 177: 2549–2562. 20864679 10.2353/ajpath.2010.100265 PMC2966811

[93] Yasuno F, Kosaka J, Ota M, Higuchi M, Ito H, et al (2012) Increased binding of peripheral benzodiazepine receptor in mild cognitive impairment-dementia converters measured by positron emission tomography with [(1)(1)C]DAA1106. Psychiatry research 203: 67–74. 22892349 10.1016/j.pscychresns.2011.08.013

[94] Kumar A, Muzik O, Shandal V, Chugani D, Chakraborty P, et al (2012) Evaluation of age-related changes in translocator protein (TSPO) in human brain using (11)C-[R]-PK11195 PET. Journal of neuroinflammation 9: 232. 23035793 10.1186/1742-2094-9-232 PMC3546876

[95] Priller C, Bauer T, Mitteregger G, Krebs B, Kretzschmar HA, et al (2006) Synapse formation and function is modulated by the amyloid precursor protein. The Journal of neuroscience: the official journal of the Society for Neuroscience 26: 7212–7221. 16822978 10.1523/JNEUROSCI.1450-06.2006 PMC6673945

[96] Mariathasan S, Newton K, Monack DM, Vucic D, French DM, et al (2004) Differential activation of the inflammasome by caspase-1 adaptors ASC and Ipaf. Nature 430: 213–218. 15190255 10.1038/nature02664

[97] Huising MO, Stet RJ, Savelkoul HF, Verburg-van Kemenade BM (2004) The molecular evolution of the interleukin-1 family of cytokines; IL-18 in teleost fish. Developmental and comparative immunology 28: 395–413. 15062640 10.1016/j.dci.2003.09.005

[98] Tay A, Simon JS, Squire J, Hamel K, Jacob HJ, et al (1995) Cytosolic phospholipase A_2_ gene in human and rat: chromosomal localization and polymorphic markers. Genomics 26: 138–141. 7782073 10.1016/0888-7543(95)80093-2

[99] Gutierrez EG, Banks WA, Kastin AJ (1994) Blood-borne interleukin-1 receptor antagonist crosses the blood-brain barrier. Journal of neuroimmunology 55: 153–160. 7829665 10.1016/0165-5728(94)90005-1

[100] Heneka MT, Kummer MP, Stutz A, Delekate A, Schwartz S, et al (2013) NLRP3 is activated in Alzheimer's disease and contributes to pathology in APP/PS1 mice. Nature 493: 674–678. 23254930 10.1038/nature11729 PMC3812809

[101] Youm Y-H, Adijiang A, Vandanmagsar B, Burk D, Ravussin A, et al (2011) Elimination of the NLRP3-ASC inflammasome protects against chronic obesity-induced pancreatic damage. Endocrinology 152: 4039–4045. 21862613 10.1210/en.2011-1326 PMC3199005

[102] Zhao Y, Kerscher N, Eysel U, Funke K (2001) Changes of contrast gain in cat dorsal lateral geniculate nucleus by dopamine receptor agonists. Neuroreport 12: 2939–2945. 11588607 10.1097/00001756-200109170-00037

[103] Stephenson DT, Lemere CA, Selkoe DJ, Clemens JA (1996) Cytosolic Phospholipase A2(cPLA2) Immunoreactivity Is Elevated in Alzheimer's Disease Brain. Neurobiology of disease 3: 51–63. 9173912 10.1006/nbdi.1996.0005

[104] Moses GSD, Jensen MD, Lue L-F, Walker DG, Sun AY, et al (2006) Secretory PLA2-IIA: a new inflammatory factor for Alzheimer's disease. Journal of neuroinflammation 3: 28. 17026770 10.1186/1742-2094-3-28 PMC1613236

[105] Rao JS, Harry GJ, Rapoport SI, Kim H-W (2009) Increased excitotoxicity and neuroinflammatory markers in postmortem frontal cortex from bipolar disorder patients. Molecular psychiatry 10.1038/mp.2009.47 PMC2844920 19488045

[106] Casey BJ, Giedd JN, Thomas KM (2000) Structural and functional brain development and its relation to cognitive development. Biological psychology 54: 241–257. 11035225 10.1016/s0301-0511(00)00058-2

[107] Morrison JH, Baxter MG (2012) The ageing cortical synapse: hallmarks and implications for cognitive decline. Nature reviews Neuroscience 13: 240–250. 22395804 10.1038/nrn3200 PMC3592200

[108] Lein ES, Hawrylycz MJ, Ao N, Ayres M, Bensinger A, et al (2007) Genome-wide atlas of gene expression in the adult mouse brain. Nature 445: 168–176. 17151600 10.1038/nature05453

[109] Loerch PM, Lu T, Dakin KA, Vann JM, Isaacs A, et al (2008) Evolution of the aging brain transcriptome and synaptic regulation. PloS one 3: e3329. 18830410 10.1371/journal.pone.0003329 PMC2553198

[110] Hawrylycz MJ, Lein ES, Guillozet-Bongaarts AL, Shen EH, Ng L, et al (2012) An anatomically comprehensive atlas of the adult human brain transcriptome. Nature 489: 391–399. 22996553 10.1038/nature11405 PMC4243026

[111] Somel M, Liu X, Khaitovich P (2013) Human brain evolution: transcripts, metabolites and their regulators. Nature reviews Neuroscience 14: 112–127. 23324662 10.1038/nrn3372

[112] Rhie SK, Coetzee SG, Noushmehr H, Yan C, Kim JM, et al (2013) Comprehensive functional annotation of seventy-one breast cancer risk Loci. PloS one 8: e63925. 23717510 10.1371/journal.pone.0063925 PMC3661550

[113] Persengiev S, Kondova I, Bontrop R (2013) Insights on the functional interactions between miRNAs and copy number variations in the aging brain. Frontiers in molecular neuroscience 6: 32. 24106459 10.3389/fnmol.2013.00032 PMC3788589

[114] Veenman L, Bode J, Gaitner M, Caballero B, Pe'er Y, et al (2012) Effects of 18-kDa translocator protein knockdown on gene expression of glutamate receptors, transporters, and metabolism, and on cell viability affected by glutamate. Pharmacogenetics and genomics 22: 606–619. 22732722 10.1097/FPC.0b013e3283544531

[115] Medzhitov R, Preston-Hurlburt P, Janeway CA Jr (1997) A human homologue of the Drosophila Toll protein signals activation of adaptive immunity. Nature 388: 394–397. 9237759 10.1038/41131

[116] Takeda K, Akira S (2005) Toll-like receptors in innate immunity. International immunology 17: 1–14. 15585605 10.1093/intimm/dxh186

