# Supplementary material for: Coordinated Gene Expression of Neuroinflammatory and Cell Signaling Markers in Dorsolateral Prefrontal Cortex during Human Brain Development and Aging
Source: PLoS One. 2014 Oct 20;9(10):e110972. doi: 10.1371/journal.pone.0110972 (PMC4203852; doi:10.1371/journal.pone.0110972)
Supplement: Table S1 — Selected genes, chromosomal locations, protein description, and major reported functions. Based on the literature, genes whose protein products participate in major functions may be categorized as follows: (1) Synaptic function, SYP, DBN1, SNCA; (2) Growth and maintenance, BDNF, NGF, GAP43, PDGFA; (3) Myelin integrity, MOBP; (4) Neuroinflammation: (a) Microglial activation, CD68, TSPO, TLR4, TLR2, NOS2, AIF1; (b) Cytokine and chemokine processes, CX3CR1, CX3CL1, IFI16, IL1B, IL1R1, IL1RN, IL1RAP, IL6, TNFRSF1A, TRAF6, TRAP1, MAPK14, MYD88; (c) Glial activation, GFAP; (d) Apoptosis, CASP1; (5) Arachidonic acid cascade, PLA2G4A, PLA2G10, PTGS1, PTGS2; (5) Amyloid membrane processing, APP, BACE; (6) Microtubules, MAP2; (7) Transcription factors, NFKB1, TFAP2A [61], [62], [64], [114]. (DOCX) [file pone.0110972.s002.docx]

Table S1. Selected genes and their chromosomal locations, proteins and

functions.

| **Gene name** | **Location** | **Protein name** | **Functions** |
| --- | --- | --- | --- |
| *AIF1* | 6p21.3 | Allograft inflammatory factor- 1 | Anti-inflammatory responses, induced by cytokines and interferon |
| *APP* | 21q21.2 | Amyloid precursor protein | Cell surface receptor and transmembrane precursor protein; AD |
| *BACE1* | 11q23-q24 | Beta-site APP-cleaving enzyme 1 | Proteolytic cleavage of APP to Aβ |
| *BDNF* | 11p14.1 | Brain derived neurotrophic factor | Survival of striatal neurons in the brain, growth, and cell differentiation |
| *CASP1* | 11q23 | Caspase 1, apoptosis-related cysteine peptidase | Inflammasome assembly, and execution-phase of cell apoptosis |
| *CD68* | 17p13 | Cluster of Differentiation 68 | Highly expressed by human monocytes and tissue macrophages.  Microglia marker in the brain, clear cellular debris, promote phagocytosis, and mediate the recruitment and activation of macrophage |
| *CX3CR1* | 3p21.3 | Chemokine (C-X3-C motif) receptor 1; (fractalkine receptor) | Adhesion and migration of leukocytes, crosstalk between microglia and neurons in the brain, expressed in microglia |
| *CX3CL1* | 16q13 | Small Inducible Cytokine Subfamily D (Cys-X3-Cys), Member 1  (fractalkine, neurotactin) | Highly expressed in neurons. regulates normal microglia function and neuron-microglia communication. |
| *DBN1* | 5q35.3 | Drebrin 1 | Process of neuronal growth, postsynaptic marker |
| *GAP43* | 3q13.31 | Growth associated protein 43 | Neuronal growth during development and axonal regeneration |
| *GFAP* | 17q21 | Glial fibrillary acidic protein | Major intermediate filament proteins of mature astrocytes. Astrocyte marker |
| *IFI-16* | 1q22 | Interferon, gamma-inducible protein 16 | Modulates p53 function, and inhibits cell growth in the Ras/Raf signaling pathway |
| *IL-1β* | 2q14 | Interleukin 1 beta | Important mediator of the inflammatory response, cell proliferation, differentiation, and apoptosis. Induction of COX-2 in the CNS contribute to inflammatory pain |
| *IL-1R1* | 2q12 | Interleukin 1 receptor, type I | Receptor involved in many cytokine-induced immune and inflammatory responses |
| *IL-1RAP* | 3q28 | Interleukin 1 receptor accessory protein | Necessary part of the interleukin 1 receptor complex |
| *IL-1RN* | 2q14.2 | Interleukin 1 receptor antagonist | Inhibits activities of interleukin 1, alpha (IL1A) and interleukin 1, beta (IL1B), and modulates a variety of interleukin 1 related immune and inflammatory responses |
| *IL-6* | 7p21-p15 | Interleukin 6 | Functions in acute and chronic inflammation, and the maturation of B cells |
| *MAP2* | 2q34-q35 | Microtubule-associated protein 2 | Microtubule assembly and neurogenesis |
| *MAPK14* | 6p21.3-p21.2 | Mitogen-activated protein kinase 14 | Wide variety of cell processes such as proliferation, differentiation, transcription regulation and development. Induced by proinflammatory cytokines |
| *MOBP* | 3p21.33 | Myelin-associated oligodendrocyte basic protein | Stabilizing the myelin sheath |
| *MYD88* | 3p22 | Myeloid differentiation primary response 88 | Adapter protein involved in the Toll-like and IL-1 receptor signaling in the innate immune response |
| *NF-κB1* | 4q24 | Nuclear factor of kappa light polypeptide gene enhancer in B-cells 1 | Pleiotropic transcription factor that responds to cytokines and stress, and plays key role in regulating the immune response to infection |
| *NGF* | 1p13.1 | Nerve growth factor | Development and maintenance of the sympathetic and sensory nervous systems |
| *NOS2* | 17q11.2-q12 | Nitric oxide synthase 2, inducible (iNOS) | Produces nitric oxide (NO), which release glutamate from axon terminals and modifies neurotransmission |
| *PDGFA* | 7p22 | Platelet-derived growth factor alpha polypeptide | Growth factor that plays an essential role in the regulation of embryonic development, cell proliferation, cell migration, survival and chemotaxis |
| *PLA2G4A* | 1q25 | Phospholipase A2, group IVA, cytosolic, calcium-dependent) (cPLA_2_ IVA) | Selectively hydrolyzes arachidonyl phospholipids in sn-2 position releasing arachidonic acid |
| *PLA2G10* | 16p13.1-p12 | Phospholipase A2, group X | Catalyzes calcium-dependent hydrolysis of 2-acyl groups in 3-sn-phosphoglycerides, releasing arachidonic acid |
| *PTGS1* | 9q32-q33.3 | Prostaglandin-endoperoxide synthase 1 (cyclooxygenase 1, COX-1) | Role in regulating or promoting cell proliferation. Prostaglandin and thromboxane production |
| *PTGS2* | 1q25.2-q25.3 | Prostaglandin-endoperoxide synthase 2 (cyclooxygenase 2, COX-2) | Major mediator of inflammation of prostanoid signaling in activity-dependent plasticity. Mediates formation of prostaglandins from arachidonate |
| *RELA* | 11q13 | V-rel avian reticuloendotheliosis viral oncogene homolog A | Formation of the NF-kappa-B complex by the Rel-like domain-containing proteins |
| *SNCA* | 4q21.3-q22 | Synuclein, alpha | Involved in regulating dopamine release and transport. Induces fibrillization of microtubule-associated protein tau |
| *SYP* | Xp11.23-p11.22 | Synaptophysin | Structural functions as organizing other membrane components and targeting synaptic vesicles to the plasma membrane. Presynaptic marker |
| *TFAP2A* | 6p24.3 | Transcription factor AP-2 alpha (activating enhancer binding protein 2 alpha) | Interacts with inducible viral and cellular enhancer elements to regulate transcription of selected genes |
| *TLR2* | 4q32 | Toll-like receptor 2 | Cooperates with LY96 to mediate innate immune response to bacterial lipoprotein and other cell wall components |
| *TLR4* | 9q33.1 | Toll-like receptor 4 | Cooperates with LY96 and CD14 to mediate innate immune response to bacterial lipopolysaccharide. Acts *via* MYD88, TIRAP and TRAF6, leading to NF-kappa-B activation, cytokine secretion and the inflammatory response. |
| *TNFRSF1A* | 12p13.2 | Tumor necrosis factor receptor superfamily, 1A | Activates NF-kappa B, mediates apoptosis, and functions as regulator of inflammation |
| *TRAF6* | 11p12 | TNF receptor-associated factor 6, E3 ubiquitin protein ligase | Mediates signaling from members of TNF receptor superfamily as well as Toll/IL-1 family |
| *TRAP1* | 16p13.3 | TNF receptor-associated protein 1 | Regulates cellular stress responses, mitochondrial chaperone protein |
| *TSPO* | 22q13.3 | Translocator protein (18kDa) | Interacts with some benzodiazepines, key factor in cholesterol flow into mitochondria for steroid hormone synthesis; neuroinflammatory PET marker |
